# Age, Gender, and Cancer but Not Neurodegenerative and Cardiovascular Diseases Strongly Modulate Systemic Effect of the Apolipoprotein E4 Allele on Lifespan

**DOI:** 10.1371/journal.pgen.1004141

**Published:** 2014-01-30

**Authors:** Alexander M. Kulminski, Konstantin G. Arbeev, Irina Culminskaya, Liubov Arbeeva, Svetlana V. Ukraintseva, Eric Stallard, Kaare Christensen, Nicole Schupf, Michael A. Province, Anatoli I. Yashin

**Affiliations:** 1 Center for Population Health and Aging, Duke University, Durham, North Carolina, United States of America; 2 Institute for Genome Science and Policy, Duke University, Durham, North Carolina, United States of America; 3 Social Science Research Institute, Duke University, Durham, North Carolina, United States of America; 4 Duke Cancer Institute, Duke University, Durham, North Carolina, United States of America; 5 The Danish Aging Research Center, University of Southern Denmark, Odense, Denmark; 6 Department of Clinical Genetics and Department of Clinical Biochemistry and Pharmacology, Odense University Hospital, Odense, Denmark; 7 Taub Institute for Research on Alzheimer's Disease and the Aging Brain, Columbia University Medical Center, New York, New York, United States of America; 8 Washington University School of Medicine, Division of Statistical Genomics, St. Louis, Missouri, United States of America; University of California San Diego and The Scripps Research Institute, United States of America

## Abstract

Enduring interest in the Apolipoprotein E (ApoE) polymorphism is ensured by its evolutionary-driven uniqueness in humans and its prominent role in geriatrics and gerontology. We use large samples of longitudinally followed populations from the Framingham Heart Study (FHS) original and offspring cohorts and the Long Life Family Study (LLFS) to investigate gender-specific effects of the ApoE4 allele on human survival in a wide range of ages from midlife to extreme old ages, and the sensitivity of these effects to cardiovascular disease (CVD), cancer, and neurodegenerative disorders (ND). The analyses show that women's lifespan is more sensitive to the e4 allele than men's in all these populations. A highly significant adverse effect of the e4 allele is limited to women with moderate lifespan of about 70 to 95 years in two FHS cohorts and the LLFS with relative risk of death RR = 1.48 (p = 3.6×10^−6^) in the FHS cohorts. Major human diseases including CVD, ND, and cancer, whose risks can be sensitive to the e4 allele, do not mediate the association of this allele with lifespan in large FHS samples. Non-skin cancer non-additively increases mortality of the FHS women with moderate lifespans increasing the risks of death of the e4 carriers with cancer two-fold compared to the non-e4 carriers, i.e., RR = 2.07 (p = 5.0×10^−7^). The results suggest a pivotal role of non-sex-specific cancer as a nonlinear modulator of survival in this sample that increases the risk of death of the ApoE4 carriers by 150% (p = 5.3×10^−8^) compared to the non-carriers. This risk explains the 4.2 year shorter life expectancy of the e4 carriers compared to the non-carriers in this sample. The analyses suggest the existence of age- and gender-sensitive systemic mechanisms linking the e4 allele to lifespan which can non-additively interfere with cancer-related mechanisms.

## Author Summary

Discovering genetic origins of healthspan and lifespan could lead to breakthroughs in increasing the years of healthy and long life. In this paper we characterize the association of the e4 allele of the well-studied ApoE gene with lifespan in two generations of participants of large longitudinal studies, the Framingham Heart Study and the Long Life Family Study, and investigate the role of major human diseases such as cardiovascular disease, cancer, and neurodegenerative disorders in this association. This wide range of systemic analyses is possible given the large sample with directly genotyped ApoE polymorphism available from these studies (N = 9841, with 2557 deaths). The analyses show that women's lifespan is more sensitive to the e4 allele than men's in these populations. However, the strongly adverse effect of the e4 allele is not observed for all women, but only for those 70 to 95 years old. Cardiovascular disease, cancer, and neurodegenerative disorders do not mediate the association of the e4 allele with lifespan. However, cancer, but not cardiovascular and neurodegenerative diseases, non-additively enhances this effect resulting in 4.2 years of difference in mean lifespan for the e4 allele carriers compared to the non-carriers.

## Introduction

The Apolipoprotein E (ApoE) common polymorphism (e2, e3, and e4) is one of the most studied genetic variants in humans. The interest in this polymorphism is two-fold. First, the functional diversity of the ApoE polymorphism appears to be a unique signature of humans with no coding variation in this gene even in human's closest ancestries in which the monomorphic ApoE sequence resembled human's e4 allele [1], [2]. Understanding the functional diversity of the ApoE gene, thus, can help in gaining insights on human evolution. Second, the ApoE polymorphism is of fundamental interest for geriatrics and gerontology because of its profound role in human diseases in late (post-reproductive) life and lifespan.

Most consistent associations were reported for the detrimental effect of the e4 allele on Alzheimer disease [3]–[5]. Studies also mostly documented a detrimental role of the e4 allele in cardiovascular health [6], [7] although a protective role of this allele was also reported [6], [8]. The e4 allele was associated with human lifespan and longevity in a number of studies [9]–[19]; some studies reported, however, no significant effect [20]–[22] (see also http://genomics.senescence.info/longevity). Studies of the role of the e4 allele in human longevity were mostly limited to comparing frequencies of genotypes in long-living individuals and younger controls [23], a strategy which has limitations [24]. Studies examining survival of older individuals carrying the e4 allele are rare (notably, [17], [18]). Sexual dimorphism of the ApoE gene in human survival has not been widely studied so far (see [17] and references therein).

Since the e4 allele may be involved in regulation of such common diseases in the elderly as dementia and cardiovascular diseases (CVD), it is often assumed that the detrimental effect of the e4 allele on human longevity is mediated by these diseases (e.g., [11], [14], [25]). Studies of the systemic effect of the e4 allele and major human diseases on lifespan in the same samples are rare [16], [17] primarily because they require large samples of genotyped individuals followed for a long period of time to have sufficient number of events.

Despite the detrimental role of the e4 allele in human health and longevity, this allele continues to be widespread in human population [26]. The persistence of this allele has been proposed to be a result of balancing selection implying that the e4 allele should be also evolutionarily advantageous with a beneficial role in early life [27]–[30].

In this work we examine three inter-related problems which, taken together, address the systemic role of the e4 allele in human lifespan. First, we investigate gender-specific effects of the ApoE4 allele on survival in a wide range of ages starting from midlife to extreme old ages. Second, we examine whether major human diseases such as CVD, cancer, and neurodegenerative disorders (ND) can explain (i.e., mediate) the effect of the e4 allele on survival. Third, we investigate whether these diseases can modulate the e4-specific survival non-additively. This wide range of systemic analyses is possible given the large sample with directly genotyped ApoE polymorphism available for the analyses and selected from the Framingham Heart Study (N = 5182) and the Long Life Family Study (N = 4659) followed longitudinally for up to 60 years with a total of 2557 deaths.

## Results

The proportions of the ApoE4 allele carriers (see Methods) and the allele-specific proportions of deaths, CVD, cancer, and ND are given in Table 1. Table 1 shows that the proportion of the e4 allele carriers is about the same regardless of gender in FHS and FHSO cohorts at the time of biospecimens collection, i.e., 20.6% FHS men, 22.3% FHSO men, 22.8% FHS women, and 22.7% FHSO women carry the e4 allele. The FHSO sample of genotyped survivors (mean age of about 50 years) was, however, about 20 years younger than that in the FHS at the time of biospecimens collection (Table 1) indicating no strong e4-specific survival selection by that time in the FHS and FHSO survivors. The proportion of the e4 allele carriers in the LLFS was the largest in spouses; it was (significantly [31]) smaller in children of the long-living individuals compared to spouses; it declined in the selected population of long-living individuals compared to younger populations.

**Table 1:** Proportions of the ApoE4 allele carriers, mean age at the time of biospecimens collection, and the allele-specific proportions of deaths, CVD, cancer, and ND for the genotyped participants of the FHS, FHSO, and LLFS.

Study	e4	N (%*)	Age (SD) years***	Death		CVD		Cancer		ND	
				N	%	N	%	N	%	N	%
***Men***											
FHS	no	362**	73.1 (5.4)	322	89.0	260	71.8	151	41.7	77	21.3
	yes	94 (20.6)	74.5 (5.6)	85	90.4	66	70.2	33	35.1	26	28.3
FHSO	no	1456	51.8 (10.2)	346	23.8	451	31.0	377	25.9	19	1.3
	yes	418 (22.3)	52.5 (10.1)	111	26.6	148	35.4	97	23.2	17	4.2
LLFS_P	no	567	90.0 (5.7)	276	48.7						
	yes	95 (14.4)	88.3 (5.2)	46	48.4						
LLFS_O	no	778	60.6 (8.4)	23	3.0						
	yes	203 (20.7)	60.3 (7.8)	3	1.5						
LLFS_S	no	333	65.1 (10.4)	23	6.9						
	yes	116 (25.8)	65.3 (9.7)	9	7.8						
***Women***											
FHS	no	619**	74.2 (5.7)	494	79.8	392	63.3	194	31.3	146	23.9
	yes	183 (22.8)	73.8 (5.7)	155	84.7	94	51.4	48	26.2	66	36.9
FHSO	no	1584	51.5 (10.1)	203	12.8	280	17.7	317	20.0	15	1.0
	yes	466 (22.7)	51.1 (9.3)	81	17.4	99	21.2	86	15.5	12	2.6
LLFS_P	no	636	91.0 (6.9)	287	45.1						
	yes	86 (11.9)	89.3 (5.7)	30	34.9						
LLFS_O	no	1069	60.4 (8.3)	23	2.2						
	yes	271 (20.2)	61.0 (7.7)	10	3.7						
LLFS_S	no	379	65.0 (13.6)	19	5.0						
	yes	126 (25.0)	64.4 (13.3)	11	8.7						

FHS = the Framingham Heart Study (FHS) original cohort; FHSO = the FHS offspring cohort.

LLFS_P = long-living parental generation of the Long Life Family Study (LLFS) participants; LLFS_O = offspring of the LLFS long-living participants; LLFS_S = spouses of the LLFS long-living participants and their offspring.

CVD = cardiovascular diseases including diseases of heart and stroke; Cancer = all sites but skin; ND = dementia and Alzheimer disease combined; SD = standard deviation.

* proportion of the ApoE4 allele carriers is in percentages;

** maximal sample size; the number of individuals with non-missing information on ND is about 1% less in the FHS and about 3% less in the FHSO.

*** age at biospecimens collection at the 19^th^ FHS, 4^th^ FHSO and baseline LLFS examinations.

The ApoE4-allele-specific proportions of CVD, cancer, and ND are not given for the LLFS because this information was not used in this paper.

### Empirical Age Patterns of Survival of the FHS and LLFS Men and Women

**Figure 1:**
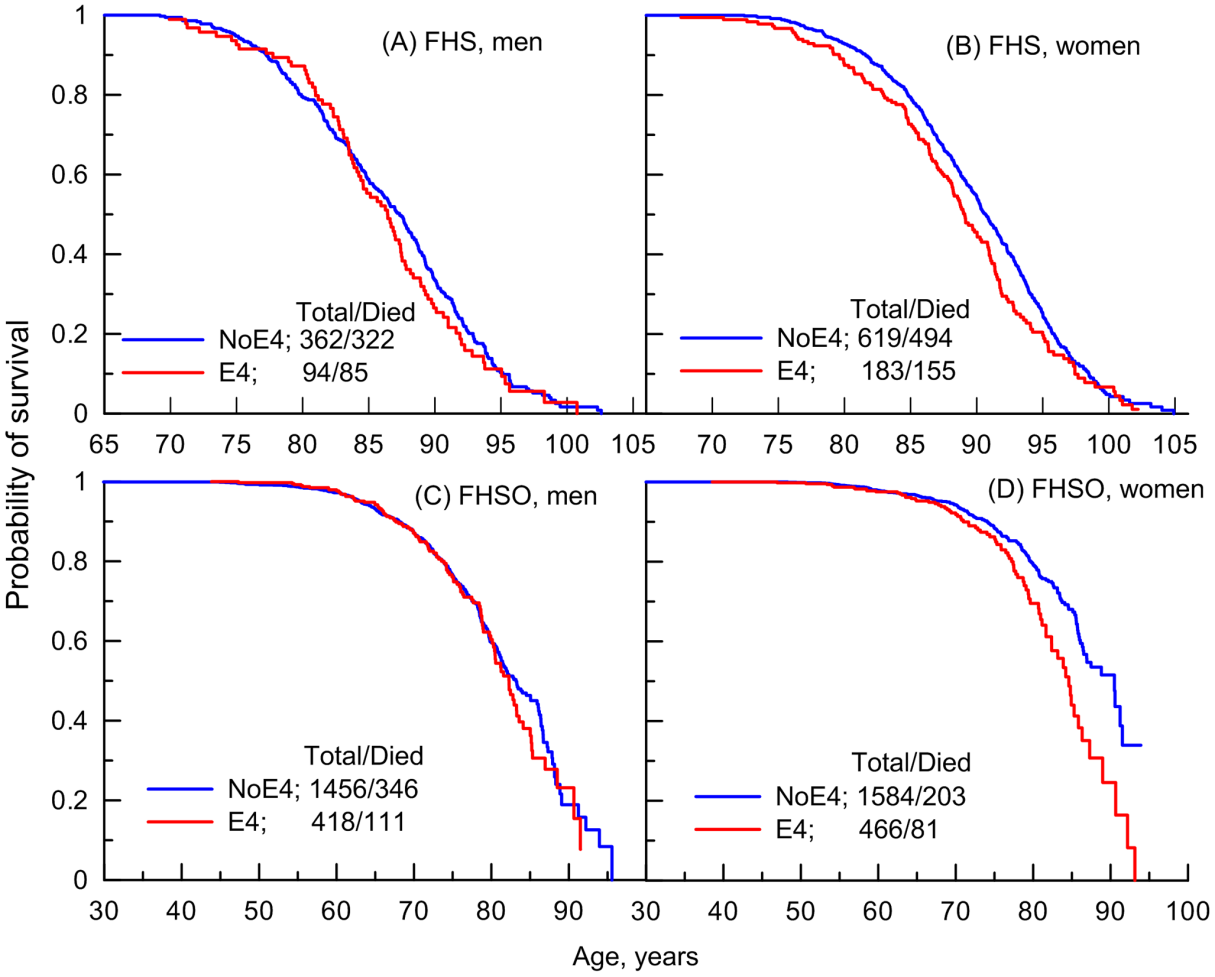
Empirical age patterns of survival of the ApoE4 carriers and non-carriers in the FHS. Patterns are shown for (A and C) men and (B and D) women genotyped in (A and B) FHS and (C and D) FHSO cohorts who carry (E4) and do not carry (NoE4) the ApoE4 allele. The numbers in the insets show the total number of genotyped individuals and the number of deaths among them.

Our empirical analysis showed no consistent detrimental effect of the e4 allele across ages on the survival of men either in the FHS or FHSO cohorts (Figures 1A and 1C). Contrary to men, the e4 female carriers have shorter lives than the non-carriers (Figures 1B and 1D). An important result is that the role of the e4 allele in survival can change with age. Specifically, there is no e4-specific difference in survival of either the FHS men or women at ages 95 years and older (Figures 1A and 1B). The e4 allele does not affect survival at ages 70 years and younger (Figure 1D) either.

**Figure 2:**
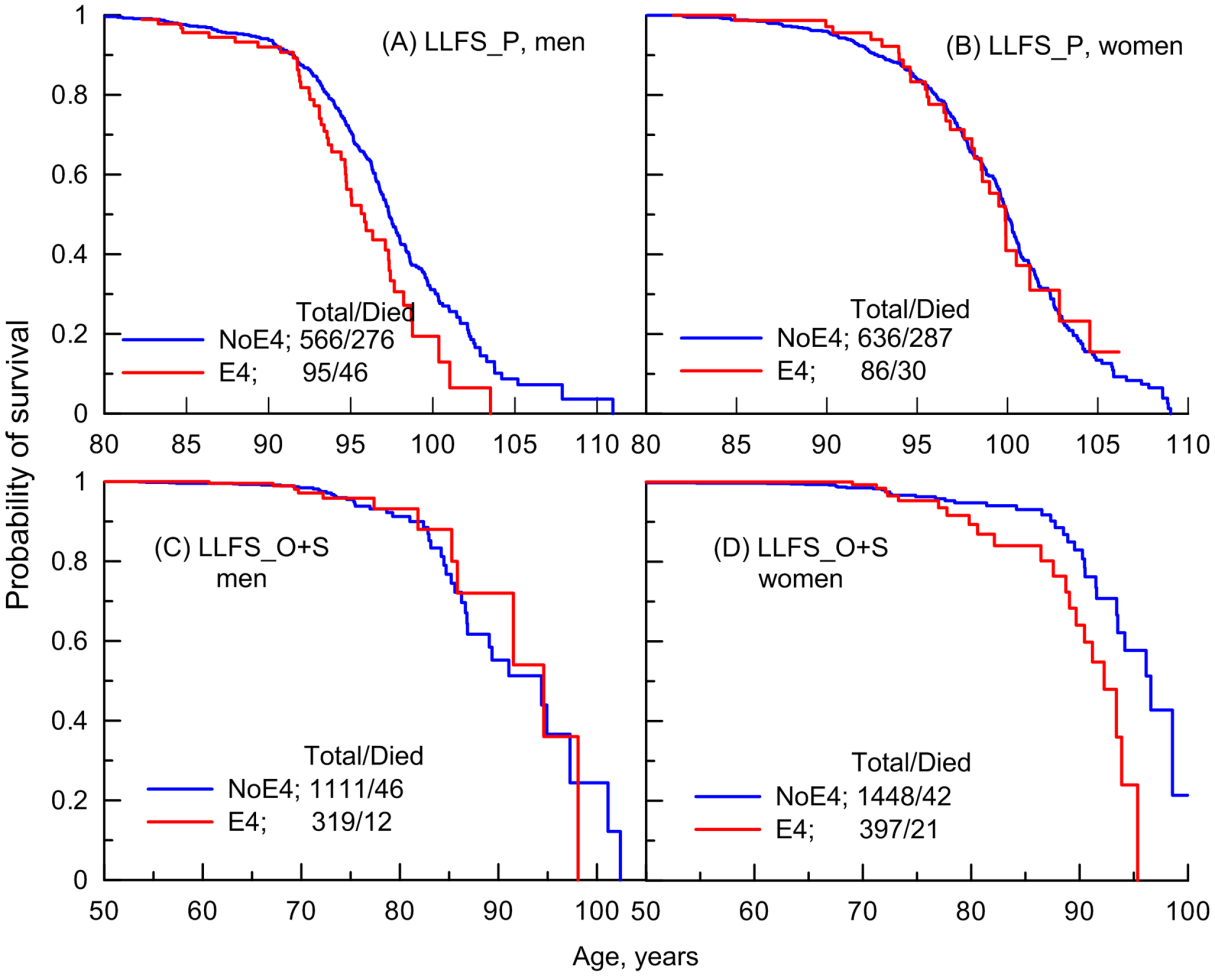
Empirical age patterns of survival of the ApoE4 carriers and non-carriers in the LLFS. Patterns are shown for (A and C) men, (B and D) women; and for (A and B) long-living individuals (LLFS_P) and (C and D) offspring of long-living individuals and spouses (LLFS_O+S) who carry (E4) and do not carry (NoE4) the ApoE4 allele. By design, the LLFS included long-living individuals who were aged 80+ years at entry. Offspring of long-living individuals and spouses were pooled together because of the small number of deaths among them (Table 1). The numbers in the insets show the total number of genotyped individuals and the number of deaths among them.

Analysis of survival age patterns of the LLFS male and female offspring/spouses directly supports these observations. Specifically, the LLFS female offspring and spouses carrying the e4 allele show worse survival than those who do not carry this allele (Figure 2D). Survival of the LLFS male offspring and spouses is not sensitive to this allele (Figure 2C).

To better understand survival age patterns of the LLFS participants from the parental generation (Figures 2A–B), one should keep in mind that this is a population selected for its exceptional chances to live a long life based on family history and their own survival to old ages (see Methods). Accordingly, this population resembles the subpopulation of individuals who survive to the very old ages in the FHS original cohort rather than the entire sample of a normal population in this cohort. Then, an important result is that the LLFS women selected for their chances of exceptional longevity (Figure 2B) and the long-living women in the FHS original cohort (represented in Figure 1B by a tail of the survival age pattern) have the same lifespan regardless of whether they carry the e4 allele. When analyzing survival age patterns one should also consider the possibility of survival selection in aging cohorts; if this selection is sensitive to a specific genetic variant then we may have biased empirical age patterns for carriers of genotypes from this variant particularly at advanced ages. Then, although the lifespans of the long-living LLFS men may be sensitive to the e4 allele (Figure 2A), further analyses are necessary (see next subsection) to determine whether this effect is real.

Thus, Figures 1B and 2B document an important result that survival of long-living women participating in the FHS (see upper tail in Figure 1B) and LLFS is insensitive to the e4 allele. Figures 1D and 2D show another remarkable result that the effect of the e4 allele on survival in the FHSO and LLFS offspring/spouses is pronounced: (i) starting at the same age, 70 years and (ii) in women only.

### Risks of Death of the FHS and LLFS Men and Women

We evaluated the sensitivity of the survival of the long-living LLFS men to the e4 allele seen in Figure 2A. Evaluation of the relative risk (RR) of death for the e4 allele carriers using a model without adjustment for birth cohorts supported the presence of the effect (RR = 1.52, p = 6.9×10^−3^) in this sample (Table 2). However, adjustment for birth cohorts entirely explained this association (RR = 1.17, p = 0.319; Table 2), suggesting that this sensitivity was likely due to differential survival of the e4 carriers and non-carriers in different birth cohorts in the LLFS.

**Table 2:** Relative risks of death for the ApoE4 allele carriers compared to the non-carriers in the selected age groups of the genotyped participants of the FHS original, FHSO, and LLFS cohorts.

Cohort	Age group	N_total_	N_died_	RR	p	95% CI
***Men***						
FHS	All	456	407	1.16	0.239	0.91–1.48
FHSO	All	1874	457	1.08	0.492	0.87–1.33
FHS+FHSO	All	2330	864	1.12	0.178	0.95–1.31
LLFS_P^*^	All	661	322	1.52	6.9×10^−3^	1.12–2.06
LLFS_P	All	661	322	1.17	0.319	0.86–1.60
LLFS_O+S	All	1430	58	0.81	0.537	0.41–1.59
FHS	≥95	29	22	2.00	0.214	0.67–5.96
FHS	<95	427	385	1.18	0.195	0.92–1.53
FHSO	≥70	892	277	1.13	0.365	0.87–1.48
FHSO	<70	982	180	0.97	0.831	0.71–1.32
FHS+FHSO	≥70–<95	1319	662	1.17	0.096	0.97–1.40
LLFS_O+S	≥70	484	44	0.64	0.294	0.28–1.47
LLFS_O+S	<70	946	14	1.20	0.328	0.37–3.86
***Women***						
FHS	All	802	649	1.25	2.7×10^−2^	1.03–1.52
FHSO	All	2050	284	1.59	2.4×10^−4^	1.24–2.05
FHS+FHSO	All	2852	933	1.36	1.3×10^−4^	1.16–1.60
LLFS_P^*^	All	722	317	0.98	0.924	0.66–1.45
LLFS_P	All	722	317	0.78	0.279	0.50–1.22
LLFS_O+S	All	1845	63	2.23	5.2×10^−3^	1.27–3.90
FHS	≥95	126	90	0.94	0.794	0.57–1.55
FHS	<95	676	559	1.37	1.7×10^−3^	1.12–1.66
FHSO	≥70	987	188	1.80	1.3×10^−4^	1.33–2.43
FHSO	<70	1063	96	1.10	0.638	0.74–1.65
FHS+FHSO	≥70–<95	1663	747	1.48	3.6×10^−6^	1.26–1.75
LLFS_O+S	≥70	596	50	3.04	7.8×10^−4^	1.59–5.81
LLFS_O+S	<70	1249	13	0.40	0.394	0.05–3.28

RR = relative risk; CI = Confidence interval; N_total_ and N_died_ denote the total number of genotyped individuals and the number of deaths among them, respectively.

All models were adjusted for birth cohorts measured as a continuous variable except the model for long-living men and women from the LLFS parental generation (LLFS_P) denoted by asterisk (^*^). Models for pooled samples in the FHS (i.e., the FHS original and FHSO cohorts; denoted as FHS+FHSO) and LLFS (i.e., the LLFS offspring and spouses; denoted as LLFS_O+S) were adjusted for potential cohort differences. Models for the LLFS were also adjusted for potential field center differences.

“All” in column “Age group” denotes the sample of all ages; other notations in this column indicate the range of ages at death or the end of follow up in each sample. For example, “≥70” implies a group of individuals who died at 70+ years or was aged 70+ years at the end of follow up.

The relative risks obtained from the data in Figures 1 and 2 revealed the presence of a significant detrimental effect of the e4 allele on survival in women in the FHS (RR = 1.25, p = 0.027), FHSO (RR = 1.59, p = 2.4×10^−4^), and LLFS offspring/spouse (LLFS_O+S; RR = 2.23, p = 5.2×10^−3^) samples (Table 2, all). No significant effect was seen in men in either sample or in long-living women in the LLFS (Table 2, all). Pooled data from the FHS and FHSO slightly improved the significance of the estimates for women, RR = 1.36, p = 1.3×10^−4^ (Table 2, FHS+FHSO, all).

However, given the empirical evidence on the substantial role of age-related heterogeneity (Figures 1 and 2), analyses of the relative risks using the Cox proportional hazards regression model, which disregards such heterogeneity, likely underestimate the effects. A more appropriate way to address the impact of age-related heterogeneity is to consider more homogeneous groups of individuals for whom the variation of the hazards is proportional over age. Empirical evidence from independent FHS and LLFS cohorts (Figures 1 and 2) suggests selecting more homogeneous groups of individuals who died or were censored at ages: (i) younger than 95 years in the FHS (note that there were virtually no genotyped individuals with lifespans less than 70 years in this sample), (ii) 70 years and older in the FHSO and LLFS_O+S (note, virtually all genotyped participants in these samples had lifespans less than 95 years), and (iii) 70 to 95 years in the pooled sample of the FHS and FHSO.

Table 2 shows that individuals from these more homogeneous groups in each sample are at substantially larger risk of death compared to the entire sample. For example, we observe 9% increment (from RR = 1.36 to RR = 1.48) in the risk of death in the more homogeneous 70–95 year group of the FHS and FHSO women. Correspondingly, the significance of the estimate also sharply increases from p = 1.3×10^−4^ to 3.6×10^−6^.

Importantly, the analyses also confirm the lack of a significant effect of the e4 allele on survival in the groups of individuals who did not belong to the selected more homogeneous groups (Table 2). Specifically, no significant effects were observed in: (a) the groups of individuals with lifespans less than 70 years in the FHSO and LLFS_O+S, (b) individuals with exceptional survival including the entire sample of the LLFS long-living men and women (LLFS_P), and (c) individuals who were aged 95 years and older in the FHS. The lack of significant effects cannot be explained by the sample size differences (Table 2).

### Do Cancer, CVD, and ND Explain the Association of the e4 Allele with Lifespan?

To address this question, we focused on the more homogeneous groups of participants of the FHS original and FHSO cohorts defined in the previous subsection (the LLFS sample is underpowered for such analyses) in order to diminish bias attributable to disproportionality of hazards when using the Cox regression model. Given slightly smaller samples of the FHS participants with known ND status (Table 1), these analyses were limited to individuals with missing information on ND excluded (sample sizes are provided in the respective tables along with the effect estimates).

**Figure 3:**
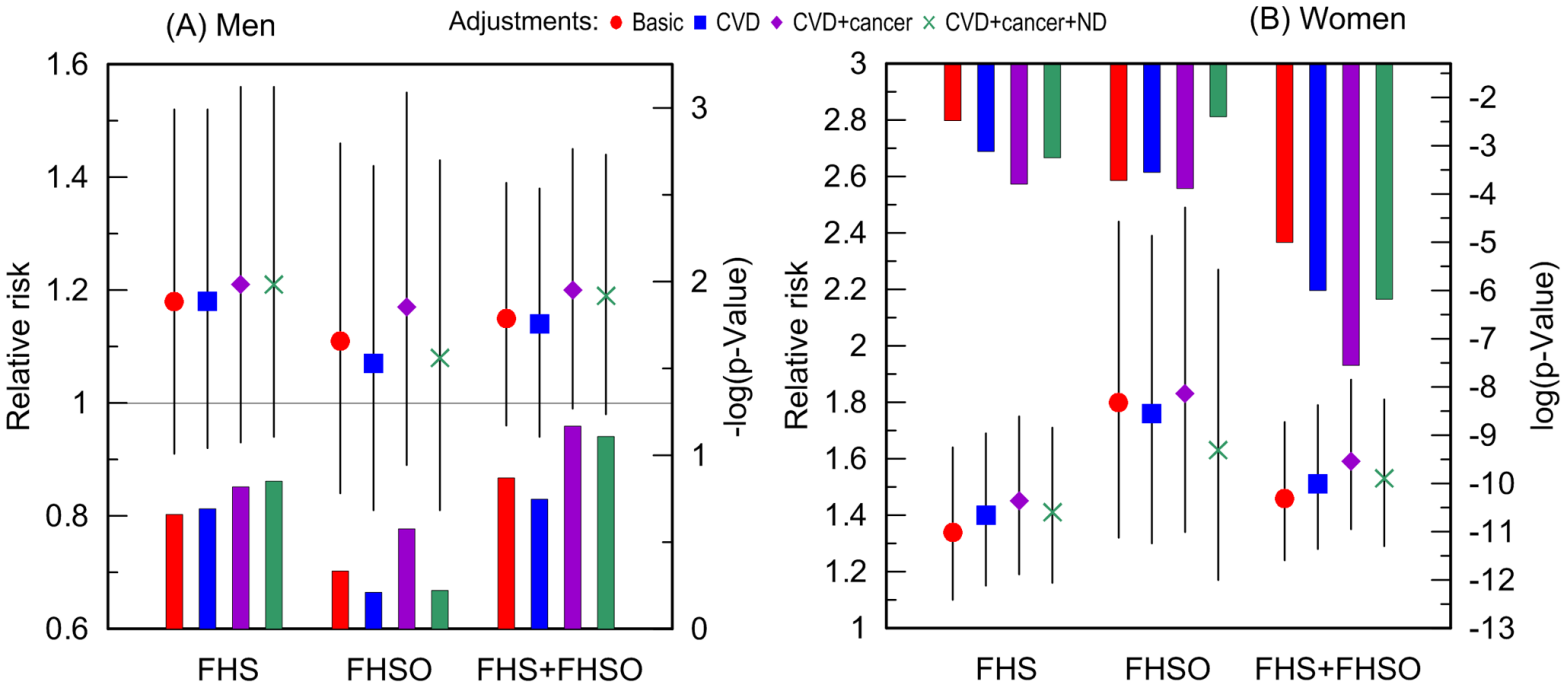
Relative risks of death and p-values for the ApoE4 allele carriers compared to the non-carriers. The risks were evaluated in more homogeneous groups of (A) men and (B) women who died or were right censored at ages: (i) younger than 95 years in the FHS, (ii) 70 years and older in the FHSO, and (iii) 70 to 95 years in the pooled sample of the FHS and FHSO cohorts. The basic model denotes adjustment for birth cohorts (all models) and an indicator of the FHS or FHSO in the pooled sample (FHS+FHSO). Adjustments by diseases are additional to the basic adjustment. Thin bars show 95% confidence intervals. Exact numeric values for the estimates and sample sizes are given in Supplementary Table S1. Right y-axes show (A) minus log-base-10-transformed p-values and (B) log-base-10-transformed p-values. (A) The horizontal line and (B) upper x-axis show the conventional level of significance, i.e., |log_10_(0.05)| = 1.3.

Additive adjustments of the Cox regression models estimating the risk of death for carriers and non-carriers of the e4 allele by (i) CVD, (ii) CVD and cancer, and (iii) CVD, cancer, and ND, reveal that CVD and cancer do not explain the observed associations. Contrarily, CVD and cancer tend to improve the estimates in each sample with a more pronounced role for cancer (Figure 3). ND plays at most minor mediating role in the associations of the e4 allele with survival of either men (Figure 3A) or women (Figure 3B). Thus, none of these diseases explain the association of the e4 allele with risks of death (see Supplementary Information, Table S1).

### Do Cancer, CVD, and ND Nonlinearly Modulate the Effect of the e4 Allele on Lifespan?

**Figure 4:**
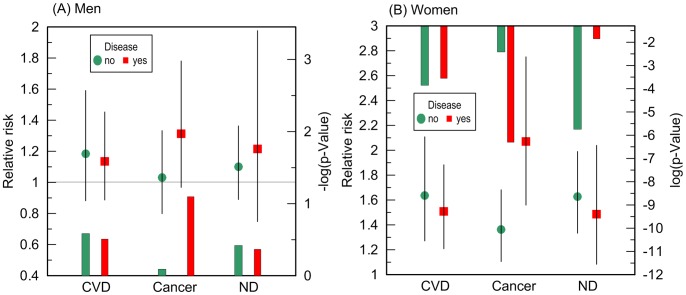
Disease-stratified relative risks of death and p-values for the ApoE4 allele carriers compared to the non-carriers. The risks were evaluated in a more homogeneous group of (A) men and (B) women who died or were right censored at ages 70 to 95 years in the pooled sample of the FHS and FHSO cohorts. The models were adjusted for birth cohorts, an indicator of the FHS or FHSO, and additive contributions of CVD, ND, and cancer, as applicable, e.g., the model for samples stratified by CVD was adjusted by cancer and ND. Multiplicative interaction between ApoE and cancer for women is significant (p = 0.029). Thin bars show 95% confidence intervals. Exact numeric values for the estimates and sample size are given in Table 3. Right y-axes show (A) minus log-base-10-transformed p-values and (B) log-base-10-transformed p-values. (A) The horizontal line and (B) upper x-axis show the conventional level of significance, i.e., |log_10_(0.05)| = 1.3.

Given no qualitative difference in the additive role of CVD, cancer, and ND in the e4-specific risks of death across the FHS samples, we evaluated the risks in the largest more homogeneous pooled sample of the FHS and FHSO participants in disease-stratified analyses (see Methods). Figure 4 and Table 3 show that the risks of death for women are the same regardless of CVD or ND status, i.e., neither CVD nor ND increase mortality of the e4 female carriers nonlinearly even after adjustment for alternative diseases. These diseases do not non-additively modulate men's survival either.

**Table 3:** Disease-stratified relative risks of death for the ApoE4 carriers compared to the non-carriers in the more homogeneous group of the FHS and FHSO participants with lifespans of 70 to 95 years.

Disease	Disease status	N_total_	N_died_	RR	p	95% CI
***Men*** *						
CVD	No	614	208	1.18	0.262	0.88–1.59
	Yes	675	442	1.14	0.313	0.89–1.45
Cancer	No	810	355	1.03	0.810	0.80–1.33
	Yes	479	295	1.31	0.080	0.97–1.78
Non-prostate	No	981	431	1.11	0.390	0.88–1.40
	Yes	308	219	1.17	0.419	0.80–1.70
ND	No	1159	527	1.10	0.376	0.89–1.36
	Yes	130	123	1.22	0.431	0.75–1.98
***Women*** *						
CVD	No	955	285	1.64	1.4×10^−4^	1.27–2.11
	Yes	680	447	1.51	2.8×10^−4^	1.21–1.88
Cancer	No	1203	475	1.36	3.8×10^−3^	1.11–1.68
	Yes	432	257	2.07	5.0×10^−7^	1.56–2.75
Non-breast	No	1360	552	1.36	1.7×10^−3^	1.12–1.66
	Yes	275	180	2.51	5.3×10^−8^	1.80–3.49
ND	No	1435	543	1.63	1.8×10^−6^	1.33–1.99
	Yes	200	189	1.49	1.4×10^−2^	1.08–2.04

* Individuals with missing neurodegenerative disorders (ND) status were excluded in all models.

CVD = cardiovascular diseases; Cancer includes all sites but skin; Non-prostate indicates non-skin cancers apart from prostate neoplasm in men; Non-breast indicates non-skin cancers apart from breast neoplasm in women;

RR = relative risk; CI = Confidence interval; N_total_ and N_died_ denote the total number of genotyped individuals and the number of deaths among them, respectively.

All models were adjusted for birth cohorts, an indicator of the FHS or FHSO, and additive contribution of CVD, ND, and cancer, as applicable, e.g., the model for samples stratified by CVD was adjusted by cancer and ND.

A striking result was that non-skin cancer significantly (p = 0.029 for multiplicative interaction of cancer with ApoE) differentiated the e4-specific risks of death for women from the more homogeneous group (with moderate lifespans of 70 to 95 years) increasing them by 52% from RR = 1.36 (p = 3.8×10^−3^) for women who did not have cancer to RR = 2.07 (p = 5.0×10^−7^) for women who had cancer (Figure 4B and Table 3). The high risk of death for women with moderate lifespan who had cancer explained the 3.2-year shorter life expectancy for the e4-allele carriers compared to the non-carriers (Table 4). The same trend on the e4-specific excess in the risks of death was seen for male cancer patients compared to non-patients (Figure 4A). Cancer increases risks for the e4 allele carriers compared to the non-carriers making them marginally significant, RR = 1.31 (p = 0.080) (Table 3).

**Table 4:** Kaplan-Meier estimates of life expectancy of the FHS and FHSO women from the more homogeneous group who were aged between 70 and 95 years at death or the end of follow up in 2008 stratified by cancer and the ApoE4 statuses.

Cancer type	Cancer status	E4 allele	N_total_	N_died_	LE, years	95% CI
All sites but skin	no	no	926	353	88.3	87.8–88.8
		yes	277	122	87.1	86.3–88.0
	yes	no	346	195	86.6	85.9–87.4
		yes	86	62	83.4	82.0–84.7
All sites but skin & breast	no	no	1052	412	88.2	87.7–88.6
		yes	308	140	87.1	86.2–87.9
	yes	no	220	136	86.2	85.2–87.1
		yes	55	44	82.0	80.4–83.5

LE = life expectancy; CI = confidence interval; N_total_ and N_died_ denote the total number of genotyped individuals and the number of deaths among them, respectively.

**Figure 5:**
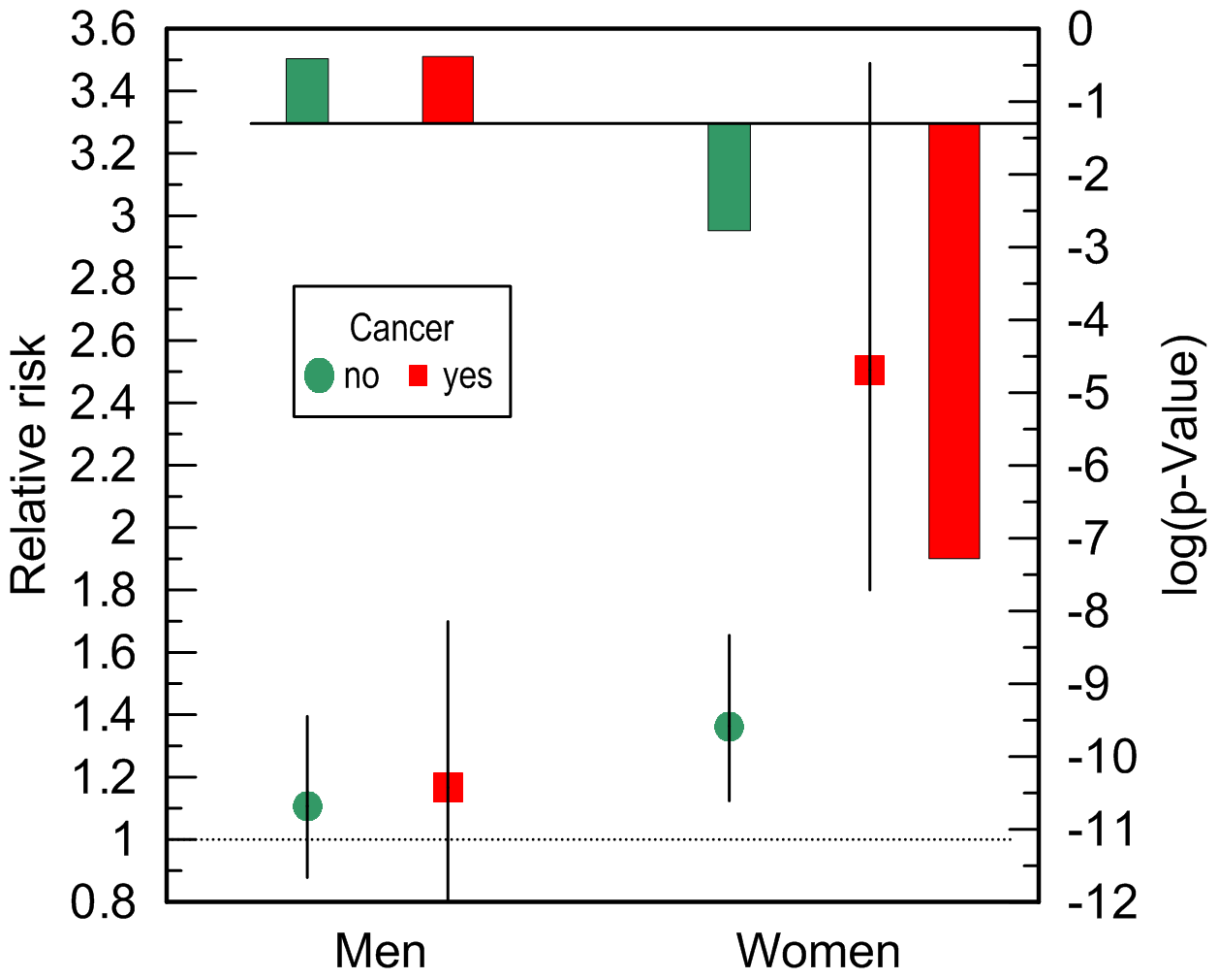
Cancer-stratified relative risks of death and log-base-10-transformed p-values for the ApoE4 allele carriers compared to the non-carriers. The risks were evaluated in more homogeneous groups of individuals who died or were right censored at ages 70 to 95 years in the pooled sample of the FHS and FHSO. “No” indicates individuals who did not have non-skin cancers apart from prostate neoplasm in men or breast neoplasm in women. “Yes” indicates individuals who had non-skin cancers other than prostate neoplasm in men or breast neoplasm in women. The models were adjusted for birth cohorts, an indicator of the FHS or FHSO, and additive contribution of CVD and ND. The multiplicative interaction between ApoE and non-sex-specific cancer in women was highly significant (p = 5.2×10^−3^). Thin bars show 95% confidence intervals. Exact numeric values for the estimates and sample size are given in Table 3 (non-prostate and non-breast). The solid horizontal line shows the conventional level of significance, i.e., log_10_(0.05) = −1.3.

The available sample size allowed us to gain some insights on potential differences between cancer sites (other than skin) in these associations. In these analyses we excluded major sex-specific sites, i.e., prostate in men and breast in women. Figure 5 and Table 3 show that relative risks of death for men without non-sex-specific cancers (RR = 1.11) increases compared to men without cancers (RR = 1.03) but it declines for men having non-sex-specific cancers (RR = 1.17) compared to men having cancers (RR = 1.31). This pattern suggests that the potential modulating effect of cancer in men is likely not sensitive to cancer site. Contrary to men, Figure 5 and Table 3 show that modulating role of cancer in women is entirely attributed to non-sex-specific cancers. The relative risk of death for women with moderate lifespan who had non-sex-specific cancers became much more pronounced (RR = 2.51, p = 5.3×10^−8^). This high risk explained the 4.2-year difference in life expectancy for the e4-allele carriers and non-carriers in this group (Table 4).

## Discussion

### The e4 Allele and Human Lifespan

Analysis of genotyped offspring in the FHS revealed that the e4 allele is irrelevant to survival in mid to early-old life, up to about 70 years (Table 2). This result appeared to be corroborated in an independent population of the LLFS offspring and spouses (Table 2). The e4 allele changed its role from neutral in mid to early-old life to detrimental at older ages. This change was found in independent samples of the FHS Offspring cohort (Figure 1D) and the LLFS offspring and spouses (Figure 2D). Moreover, this change occurred concordantly in the FHSO and LLFS: (i) at about the same age of 70 years and (ii) in women only. The detrimental effect of the e4 allele at old ages (until 95 years of age) was also found in a sample of the FHS women (Figure 1B; note that virtually no individuals with lifespan less than 70 years were genotyped in this cohort).

At extreme ages (95 years and older) we concordantly observed a neutral role of the e4 allele in each gender in the FHS (Figures 1A and 1B). Analysis of the long-living individuals in the LLFS corroborated these findings (see the “**Empirical Age Patterns of Survival of the FHS and LLFS Men and Women**” and “**Risks of Death of the FHS and LLFS Men and Women**” subsections).

Overall, these analyses demonstrated a strong detrimental effect of the e4 allele on survival which was mostly attributed to women with moderate lifespans of 70 to 95 years in the FHS, FHSO, and LLFS. For example, the e4 allele increased the risks of death of the FHS and FHSO women by about 48% (RR = 1.48) with very high confidence, p = 3.6×10^−6^ (Table 2).

Although our study provided robust evidence of a women-specific detrimental effect of the e4 allele on lifespan in three different samples of mostly North-American population (i.e., FHS, FHSO, and LLFS, see Methods), there is also robust evidence of a detrimental effect of this allele in Swedish men but not women [17]. Further, although our results on the neutral role of the e4 allele at extreme ages (95 years and older) are in agreement with some meta-analyses [e.g., 32], there is also evidence of a significant detrimental effect of the e4 allele at those ages in the Danish population [18]. The results by Rosvall et al. [17], Jacobsen et al. [18], and ours explicitly show that the effect of the e4 allele on lifespan may not be the same in different populations. These robust evidences from different populations illustrate that the concept of replication of the same effect of the same allele on the same complex phenotype characteristic for post-reproductive period has inherent limitations [33]–[36].

### The e4 Allele, Human Lifespan, and Additive Effects of Major Diseases

The e4 allele is a major susceptibility allele for Alzheimer disease (which is a subtype of the ND in this study) particularly in Caucasians [4] (but may be not in Hispanics [37]). Despite that, our well-powered analyses show that ND explains at most a tiny part in the association of the e4 allele with survival (Figure 3). The results of our analyses do not support the hypothesis that the lack of a mediating effect of ND can be due to potential ND misclassification. This is evidenced in Figure 3 by: (i) the tiny reduction of the effect size attributed to ND (Figure 3) despite the large prevalence of ND (particularly in the FHS as the older cohort, Table 1), and (ii) the role of cancer as a nonlinear modulator of the effect of the e4 allele on survival (Figure 4). Additive contributions of the e4 allele and dementia to survival was also observed in other studies [16] although the attenuation of the effect size by dementia varied [17].

Despite the associations of the e4 allele with CVD [6], [7] and with CVD-free life [19], [38], our analyses show that CVD does not explain the effect of the e4 allele on women's survival (Figure 3). Recent analyses support these results by showing independent associations of the e4 allele and various characteristics of cardiovascular health and CVD with survival [16], [17], [39].

Several studies reported on a role of the ApoE gene in cancer [40]–[43]. It has been also shown that the e4 allele can increase cancer-free lifespan in the FHS and FHSO men [19], [38]. The analyses in this study show no mediating role of cancer in the association of the e4 allele with women's survival; the additive contribution of cancer, however, can modulate the effect of the e4 allele, increasing the strength of this association (Figure 3).

CVD and cancer are the most common causes of death in humans and ND is fast growing cause of death in the elderly. CVD and ND are the diseases which have been most consistently associated with ApoE4. Despite that, these diseases do not explain the detrimental role of the e4 allele in lifespan. This finding implies the existence of a mechanism linking the e4 allele with lifespan which is largely independent of the mechanisms affecting susceptibility to CVD, cancer, and ND. Given also that the e4 allele may not be associated with frailty [14], [44], it is likely that this allele can be directly involved in regulation of human aging through intrinsic biological mechanisms. One potential mechanism could be associated with inflammation which may be involved in aging through two main pathways associated with “immunosenescence and synergies with chronic diseases that have inflammatory components” [29]. Given no mediating role of CVD, cancer, and ND observed in our study and that these diseases (particularly CVD and ND) can have e4-specific inflammatory etiology [8], [45], it might well be the case that the e4 allele affects survival through immunosenescence whereas it affects the risks of diseases through disease-specific inflammatory component.

### The e4 Allele, Human Lifespan, and Non-Additive Effects of Major Diseases

Neither CVD nor ND non-additively (i.e., nonlinearly) modulates the detrimental effect of the e4 allele on women's survival, i.e., the relative risks of death for the e4 allele carriers are the same regardless of women's CVD and ND statuses (Figure 4B). This result is in line with findings by Little et al. [16]. However, the e4 allele was shown to be mostly associated with dementia-caused deaths by Newman et al. [39].

We found that cancer showed a significant nonlinear modulating effect in the association of the e4 allele with women's survival (Figure 4B). The e4-positive female cancer patients have about a two-fold increased risk of death at ages between 70 and 95 years compared to the non-e4 allele carriers (RR = 2.07) which is highly significant, p = 5.0×10^−7^ (Table 3). Such a strong effect results in a 3.2-year shorter life expectancy of the e4 carriers compared to the non-carriers in this sample (Table 4). Further analyses show that this effect is attributed to non-sex-specific cancer sites, it substantially increases, i.e., RR = 2.51, p = 5.3×10^−8^ (Table 3), and it explains the large 4.2 year differential in the life expectancy (Table 4). Women without cancer carrying the e4 allele are still at significant risk of death. The same non-additive role of cancer was found in the effect of the e4 allele on men's survival, i.e., this allele negatively affected cancer survivorship (Figure 4A). The diminished role of cancer as a nonlinear modulator of the effect of the e4 allele on survival in men compared to women can be attributed to a protective role of this allele in susceptibility to risk of cancer in men but not in women [19], [38], i.e., protection against risks of cancer may well explain modest risks of cancer survivorship of male e4 carriers.

The cancer-sensitive non-additive effect of the e4 allele on human lifespan suggests that mechanisms associated with cancer survivorship (i.e., with its progression and/or treatment) can interfere with a mechanism linking the e4 allele to lifespan. Our findings are particularly in line with inflammatory pathways [29], [43] which may overlap for aging and cancer survivorship as a result of the compromising of the immune system with age [46] (see also next subsection). Thus, the non-additive role of cancer in the effect of the e4 allele on lifespan and the lack of this role for CVD and ND likely underscores the synergism between cancer and aging.

### The e4 Allele and Survival in Humans

Given the persistence of the e4 allele in humans, it may be beneficial in early life and, thus, be subject to balancing selection [27]–[30]. Indeed, several studies provided support for a beneficial role of the e4 allele in early life. For example, it was shown that the proportion of the e4 allele was significantly smaller in spontaneously aborted embryos than in adults [47]. The proportion of the e4 allele was also found to be significantly larger in healthy liveborn infants compared with stillborn infants and with adults [48]. These findings suggest that the e4 allele can benefit early survival. Then, given the detrimental role of this allele for survival in old ages, we should expect a neutral role at some point in between. Our finding of a neutral role of the e4 allele in survival in mid to early-old life of the genotyped FHS and LLFS participants supports this logic.

Studies also show that ApoE4 may protect against early life infectious diseases such as, e.g., diarrhea [49] and liver damage caused by the hepatitis C virus infection [50], [51]. A putative protective mechanism may be associated with an enhanced function of the immune system in early life [25] with a role of ApoE as an immunomodulator [52]. At old ages immunosenescence may be a factor favoring neoplasia [53]. Then, if ApoE4 boosts the immune system in early life, this may naturally lead to prematurely exhausting this system later in life which may affect cancer survivorship for carriers of this allele (and, thus, implying antagonistic pleiotropy). This hypothesis is supported by our findings of a strong non-additive modulating role of cancer in survival of female e4 allele carriers (Figure 5), by the very high proportion of deaths (80%) among female e4 carriers with non-sex-specific cancer by age 95 years (44 deaths among 55 carriers; Table 4), and by the 150% excess risk of death for such women compared to the non-e4 carriers (RR = 2.51, p = 5.3×10^−8^; Table 3). These high death rates can, in part, explain the diminishing detrimental effect of ApoE4 at very advanced ages (95+ years) in the FHS.

The lack of an association of ApoE4 with survival at extreme ages (95+) in the FHS and in an exceptional population of the LLFS long-living participants suggests that the detrimental effect of ApoE4 can be counterbalanced in some individuals. Potential factors can include buffering mechanisms (by other genes [54]) and/or environmental modulations of genetic effects [36]. Given large samples of long-living individuals in the LLFS, this study could be highly promising for revealing such mechanisms.

### Concluding Remarks

Analyses of the association of the ApoE4 allele with lifespan in three populations of the FHS, FHSO, and LLFS participants showed that women's lifespan was more sensitive to the e4 allele than men's. The adverse role of the e4 allele was limited to women with moderate lifespans of about 70 to 95 years; no survival disadvantage is seen for women with lifespans less than 70 or more than 95 years. The highly significant association of the e4 allele with lifespan was not explained by major diseases including CVD, ND, and cancer, whose risks can be sensitive to this allele, in large FHS samples. Non-skin cancer non-additively increased mortality of the FHS women with moderate lifespans increasing the risks of death of the e4 carriers two-fold compared to the non-carriers. High and highly significant risks of death of the e4-allele carriers in this sample explained their 3.2 year shorter life expectancy. The results suggest a pivotal role of non-sex-specific cancer as a nonlinear modulator of survival in this sample of women that increased the risk of death of the ApoE4 carriers by 150% (p = 5.3×10^−8^) compared to the non-carriers and explained the 4.2 year differential in life expectancy in this group. Our results suggest the existence of age- and gender-sensitive systemic mechanisms linking the e4 allele to lifespan which can non-additively interfere with cancer-related mechanisms.

## Methods

### Data

#### The Framingham Heart Study (FHS)

The original (FHS) cohort was launched in 1948 in Framingham, Massachusetts. 22 years later a cohort of offspring of participants of the FHS original cohort was launched (known as the FHS Offspring or FHSO cohort). The study design has been previously described [55]–[57]. Briefly, the FHS includes N = 5,209 respondents aged 28–62 years at baseline who have been biennially followed during nearly 60 years. The FHSO respondents (N = 5,124) aged 5–70 years at baseline were biological descendants (N = 3,514), their spouses (N = 1,576), and adopted offspring (N = 34) of the FHS participants who have been examined about every four years at eight visits.

The FHS/FHSO participants have been followed for the onset of CVD, cancer, and death through regular examinations at the FHS clinic, surveillance of hospital admissions, and death registries since baseline examinations in the FHS and FHSO [55], [56], currently through 2008. Dementia-free survivors who attended examination 14 in the FHS and examination two in the FHSO were continuously followed for onset of dementia and Alzheimer disease [58], currently through 2008.

Biospecimens were mostly collected in the late 1980s, and through 1990s, from surviving participants [59], [60]. The procedure used for the ApoE genotyping was described in Lahoz et al. [59]. The data available for this study include information on the ApoE2/3/4 polymorphism for the 1,258 FHS and 3,924 FHSO participants.

#### The Long Life Family Study (LLFS)

The LLFS collected data in about equal proportions at four field centers (three in the U.S., i.e., Boston, New York, and Pittsburg, and one in Denmark) on families showing exceptional familial longevity (virtually all participants were whites). The study eligibility criteria were described elsewhere [61]–[63].

Briefly, in the U.S., the families eligible for the LLFS must have two living siblings aged 80+ years, two living offspring of one or more of the siblings, and a living spouse of one of the offspring who were considered as controls. In addition, the family must demonstrate exceptional longevity based on a Family Longevity Selection Score, which is a summary-measure based on the survival experience of the oldest living generation of siblings relative to what would be expected based on birth cohort life tables [61]. Families with members of this generation who were still alive and larger sibships were given higher priorities. Finally, an eligible family was enrolled in the LLFS if at least 3 family members (the proband, at least one sibling of the proband, and one offspring of the proband or the sibling) indicated their willingness to participate.

In Denmark, individuals who would be aged 90+ years during the study recruitment period were first identified in the Danish National Register of Persons [62]. Then, using information on the place of birth and the names, parish registers available in regional archives were searched to locate the parents of the elderly individuals in order to identify sibships. The identified subjects were contacted to further assess the family's eligibility for participation in the LLFS using criteria parallel to that used in the U.S.

Information from the 4,954 U.S. and Danish LLFS participants was collected using similar questionnaires and in-home physical examinations at baseline between 2006 and 2009. Once enrolled, the LLFS participants were followed longitudinally. During the follow up for about 6 years (currently through April 2013) self-reported information on diseases collected at baseline was updated and information on vital status was collected.

Biospecimens were collected at baseline. Genotyping of the ApoE polymorphism was conducted using procedures detailed elsewhere [31]. The data include information on the ApoE2/3/4 polymorphism for the 4,659 LLFS participants including long-living individuals (N = 1,384, probands and siblings), their offspring (N = 2,321, children, nieces, and nephews), spouses of long-living individuals (N = 177), and spouses of offspring of long-living individuals (N = 777). Due to small numbers of spouses of the long-living individuals, they were pooled together with spouses of offspring (N = 954).

### Analysis

We use data on longitudinally followed FHS/FHSO and LLFS participants to characterize the role of the ApoE4 allele (e2/4, e3/4, and e4/4) and non-e4 genotypes (e2/2, e2/3, and e3/3) in the lifespans of men and women separately.

Associations of the e4 allele with risks of death were characterized by the Kaplan-Meier estimator and the Cox proportional hazards regression model. The time variable in the analyses was age at death or age at the end of follow up. The model adjustments were explicitly stated when applicable.

To examine whether or not major human diseases can shape the association of the e4 allele with survival, we considered additive and nonlinear roles of CVD (diseases of hearth and stroke combined), cancer, and ND (dementia and Alzheimer disease combined) in this association. We considered all non-skin cancers unless explicitly stated. CVD and ND were chosen because they were most consistently associated with the ApoE polymorphism [3], [6], [7], [64]. Recent studies also showed that the ApoE polymorphism can be associated with cancer [e.g., 41]. These analyses were conducted using rigorously ascertained information on diseases in the FHS/FHSO only because the LLFS data are currently underpowered for such analyses.

To address nonlinear effect of diseases on the association of the e4 allele with survival, we conducted disease-stratified analyses. Each disease group included individuals who were diagnosed with the disease (or died from it) prior to death or the end of follow up in 2008. Otherwise, individuals were included in the complementary non-disease group [65].

We used the robust sandwich estimator of variances in the Cox model to account for potential clustering (e.g., familial). Statistical analyses were conducted using SAS (release 9.3, Cary, NC, USA).

This study used de-identified data from the FHS and LLFS. The FHS data are available from the NHLBI through dbGaP. No new data were collected in this work. As such, this study does not require either ethics committee approval or written consent.

## Supporting Information

Table S1 Disease-conditional and unconditional relative risks of death for the ApoE4 allele carriers compared to the non-carriers in the selected age groups of the genotyped participants of the FHS original and FHSO cohorts.
(DOC)
