# Supplementary material for: Age, Gender, and Cancer but Not Neurodegenerative and Cardiovascular Diseases Strongly Modulate Systemic Effect of the Apolipoprotein E4 Allele on Lifespan
Source: PLoS Genet. 2014 Jan 30;10(1):e1004141. doi: 10.1371/journal.pgen.1004141 (PMC3907310; doi:10.1371/journal.pgen.1004141)
Supplement: Table S1 — Disease-conditional and unconditional relative risks of death for the ApoE4 allele carriers compared to the non-carriers in the selected age groups of the genotyped participants of the FHS original and FHSO cohorts. (DOC) [file pgen.1004141.s001.doc]

Table S1. Disease-conditional and unconditional relative risks of death for the ApoE4 allele carriers compared to the non-carriers in the selected age groups of the genotyped participants of the FHS original and FHSO cohorts.

| Cohort | Age group, yrs | N_total_ | N_died_ | Adjustment | RR | p-value | 95% CI |
| --- | --- | --- | --- | --- | --- | --- | --- |
| ***Men***^*^ | | | | | | | |
| FHS | <95 | 424 | 382 | Basic | 1.18 | 0.219 | 0.91-1.52 |
|  |  |  |  | CVD | 1.18 | 0.203 | 0.92-1.52 |
|  |  |  |  | CVD+cancer | 1.21 | 0.152 | 0.93-1.56 |
|  |  |  |  | CVD+cancer+ND | 1.21 | 0.141 | 0.94-1.56 |
| FHSO | ≥70 | 865 | 268 | No | 1.11 | 0.464 | 0.84-1.46 |
|  |  |  |  | CVD | 1.07 | 0.615 | 0.81-1.42 |
|  |  |  |  | CVD+cancer | 1.17 | 0.265 | 0.89-1.55 |
|  |  |  |  | CVD+cancer+ND | 1.08 | 0.600 | 0.81-1.43 |
| FHS+FHSO | ≥70 - <95 | 1289 | 650 | Basic | 1.15 | 0.135 | 0.96-1.39 |
|  |  |  |  | CVD | 1.14 | 0.179 | 0.94-1.38 |
|  |  |  |  | CVD+cancer | 1.20 | 0.068 | 0.99-1.45 |
|  |  |  |  | CVD+cancer+ND | 1.19 | 0.078 | 0.98-1.44 |
| ***Women***^*^ | | | | | | | |
| FHS | <95 | 666 | 549 | Basic | 1.34 | 3.3×10^-3^ | 1.10-1.64 |
|  |  |  |  | CVD | 1.40 | 7.6×10^-4^ | 1.15-1.69 |
|  |  |  |  | CVD+cancer | 1.45 | 1.6×10^-4^ | 1.19-1.75 |
|  |  |  |  | CVD+cancer+ND | 1.41 | 5.6×10^-4^ | 1.16-1.71 |
| FHSO | ≥70 | 969 | 183 | Basic | 1.80 | 1.9×10^-4^ | 1.32-2.44 |
|  |  |  |  | CVD | 1.76 | 2.8×10^-4^ | 1.30-2.39 |
|  |  |  |  | CVD+cancer | 1.83 | 1.3×10^-4^ | 1.34-2.49 |
|  |  |  |  | CVD+cancer+ND | 1.63 | 4.0×10^-3^ | 1.17-2.27 |
| FHS+FHSO | ≥70 - <95 | 1635 | 732 | Basic | 1.46 | 9.9×10^-6^ | 1.24-1.73 |
|  |  |  |  | CVD | 1.51 | 1.0×10^-6^ | 1.28-1.79 |
|  |  |  |  | CVD+cancer | 1.59 | 2.8×10^-8^ | 1.35-1.88 |
|  |  |  |  | CVD+cancer+ND | 1.53 | 6.6×10^-7^ | 1.29-1.81 |

^*^Individuals with missing neurodegenerative disorders (ND) status were excluded in all models.

RR=relative risk; CI=Confidence interval; N_total_ and N_died_ denote the total number of genotyped individuals and the number of deaths among them, respectively.

Basic model denotes adjustment for birth cohorts (all models) and an indicator of the FHS or FHSO in the pooled sample (FHS+FHSO). Other adjustments are additional to the basic model.
